# HO-3867 Induces Apoptosis via the JNK Signaling Pathway in Human Osteosarcoma Cells

**DOI:** 10.3390/pharmaceutics14061257

**Published:** 2022-06-13

**Authors:** Peace Wun-Ang Lu, Chia-Hsuan Chou, Jia-Sin Yang, Yi-Hsien Hsieh, Meng-Ying Tsai, Ko-Hsiu Lu, Shun-Fa Yang

**Affiliations:** 1 Morrison Academy Taichung, Taichung 406, Taiwan; lup@mca.org.tw; 2 Institute of Medicine, Chung Shan Medical University, Taichung 402, Taiwan; wishwing1109@hotmail.com (C.-H.C.); gazn_sheep@yahoo.com.tw (J.-S.Y.); hyhsien@csmu.edu.tw (Y.-H.H.); vickyfatfat5252@gmail.com (M.-Y.T.); 3 Department of Medical Research, Chung Shan Medical University Hospital, Taichung 402, Taiwan; 4 Department of Orthopedics, Chung Shan Medical University Hospital, Taichung 402, Taiwan; 5 School of Medicine, Chung Shan Medical University, Taichung 402, Taiwan

**Keywords:** apoptosis, curcumin, ERK, HO-3867, JNK, osteosarcoma

## Abstract

Metastatic osteosarcoma often results in poor prognosis despite the application of surgical en bloc excision along with chemotherapy. HO-3867 is a curcumin analog that induces cell apoptosis in several cancers, but the apoptotic effect and its mechanisms on osteosarcoma cells are still unknown. After observing the decrease in cellular viability of three human osteosarcoma U2OS, HOS, and MG-63 cell lines, and the induction of cellular apoptosis and arrest in sub-G1 phase in U2OS and HOS cells by HO-3867, the human apoptosis array showed that heme oxygenase (HO)-1 and cleaved caspase-3 expressions had significant increases after HO-3867 treatment in U2OS cells and vice versa for cellular inhibitors of apoptosis (cIAP)1 and X-chromosome-linked IAP (XIAP). Western blot analysis verified the results and showed that HO-3867 activated the initiators of both extrinsic caspase 8 and intrinsic caspase 9, and significantly increased cleaved PARP expression in U2OS and HOS cells. Moreover, with the addition of HO-3867, ERK1/2, and JNK1/2 phosphorylation were increased in U2OS and HOS cells. Using the inhibitor of JNK (JNK in 8), HO-3867’s increases in cleaved caspases 3, 8, and 9 could be expectedly suppressed, indicating that JNK signaling is responsible for both apoptotic pathways, including extrinsic and intrinsic, in U2OS and HOS cells caused by HO-3867. Through JNK signaling, HO-3867 has proven to be effective in causing both extrinsic and intrinsic apoptotic pathways of human osteosarcoma cells.

## 1. Introduction

Osteosarcoma, the most common tumor of primary malignant bone tumor, is mostly found in children and adolescents with a peak of incidence at 11–15 years and about six in every million children [1,2]. Typically, complete surgical en bloc excision, or extensive amputation of the affected area, was the most common type of treatment when attempting a complete radical excision, but it did not provide a good prognosis. Fortunately, chemotherapy has become a vital part for the treatment of osteosarcoma [3,4]. Using a combination of chemotherapy and surgery, long-term survival rates of diagnosed patients have improved to approximately 68–75% at 5 years for diagnosed patients with localized tumor [2,5]. Unfortunately, due to its metastatic ability, lung transfer osteosarcoma is still responsible for undesirable outcomes and fatalities [6,7]. To prevent this, several approaches to the development of new compounds containing anticancer mechanisms such as cytotoxic and antimetastatic activities need to be developed.

Apoptosis, characterized by several biochemical hallmarks such as chromatin condensation, membrane blebbing, cellular shrinkage, DNA fragmentation, and apoptotic body formation, is an important aspect of physiological growth control and regulation of the tissues to lead to programmed cell death [8]. To initiate apoptosis within cancer cells, initiator and effector proteins need to be stimulated through either the intrinsic or the extrinsic pathway, while the cell senses intracellular stress or signals from outside [9,10]. During apoptosis, cell signaling often affects many stress-inducible molecules and proteases such as ERK, JNK, and p38 of mitogen-activated protein kinases (MAPKs), caspases 8 and 9, as well as their downstream caspases 3 and 7 [11]. Initiator caspases 8 and 9, and effector caspases 3 and 7, contain modulators of apoptosis such as cellular inhibitors of apoptosis 1 and 2 (cIAP1 and 2) as well as X-chromosome-linked IAP (XIAP) [12,13,14]. As there are several molecules that can be monitored for its activity, apoptosis is often used as the treatment for cancer, which can be done through many scenarios [15,16,17]. However, treatment failures such as overexpression of IAP can not only potentially lead to cancer survival, disease progression, and poor prognosis, but the tumor could also potentially develop resistance to future chemotherapeutic treatments [18]. As such, the underlying mechanisms of apoptosis must be recognized to guarantee a higher rate of successful treatments.

Curcumin (diferuloylmethane), a compound harvested from *Curcuma longa* plants, uses a variety of cellular-signaling pathways to demonstrate a number of uses including anti-inflammatory, antioxidant, antiviral, antibacterial, antifungal, and anticancer mechanisms [19,20,21]. Through experiments, curcumin has proved to be cytotoxic among osteosarcoma [22,23]. Despite being safe at high doses in humans, rapid metabolism within the body leads to low absorption of curcumin, which unfortunately prevents curcumin from exercising its full potential. To improve the outcomes, multiple approaches have successfully created adjuvants and structural analogs of curcumin. HO-3867, a novel diarylidenylpieperidone (DAP)-inspired analog, possesses enhanced anticancer properties indicating its potential as a candidate for future chemotherapy treatments [18,24,25]. Although as a curcumin analog that possesses enhanced anticancer properties to its original counterpart, we lack data on the specific effects of the anticancer mechanisms that HO-3867 possesses on human osteosarcoma. Therefore, a series of in vitro experiments involving the analog as well as several human osteosarcoma cell lines were conducted to discover the underlying mechanisms of HO-3867 in terms of its apoptotic ability.

## 2. Materials and Methods

### 2.1. Cell Culture and HO-3867 Treatment

All the human osteosarcoma U2OS, HOS, and MG-63 cells were purchased from the FIRDI (Hsinchu, Taiwan). The U2OS cells were cultured in DMEM and supplemented with 10% FBS, 5 mL of glutamine, and 1% penicillin. The HOS and MG-63 cells were cultured in DMEM and supplemented with 10% FBS, 1% penicillin/streptomycin, and 5 mL glutamine. The cell cultures were maintained at 37 °C in a humidified atmosphere of a 5% CO_2_ incubator. HO-3867 was obtained from Tokyo Chemical Industry Co., Ltd. (Tokyo, Japan).

### 2.2. Microculture Tetrazolium Colorimetric (MTT) Assay

To gather data regarding the effects of apoptosis caused by HO-3867 on osteosarcoma cells, we extracted cells from 8.5 × 10^4^/well of U2OS, of HOS, and of MG-63 and applied different HO-3867 concentrations (0, 2, 4, 8, 16, and 32 μM) of for 24 h within 24-well plates. After completing the exposure period, the media was separated and the cells were washed using phosphate-buffered saline. Then, new medium was added and the cells were then incubated using MTT [26,27]. Following solubilization with isopropanol, the viable cell number, directly proportional to the production of formazan, was measured spectrophotometrically at 563 nm.

### 2.3. Flow Cytometric Analysis

We can determine the phases of the cell cycle affected by HO-3867, as well as several other cellular components such as DNA, using flow cytometry analysis on U2OS and HOS cells. Summarily, we plated 8 × 105 U2OS and 6 × 10^5^ HOS cells in 6 cm dishes and placed them in experimental concentration range (0, 2, 4, 8, and 16 μM) of HO-3867 for 24 h. After propidium iodide (PI) staining, 2 × 105 U2OS and HOS cells were placed in an Eppendorf tube to analyze the cell cycle using a BD AccuriTM C6 Plus personal flow cytometer [28,29].

### 2.4. Annexin V-FITC Apoptosis Staining Assay

After going through apoptosis, cells would translocate membrane phospholipid phosphatidylserine molecules from the inner to the surface layer of the plasma membrane. Using Annexin V, a conjugated fluorescent protein with a high affinity for the translocated molecules, we stained the phospholipid phosphatidylserine molecules, which were now exposed externally, making it easier to identify apoptosis in earlier stages than other assays, such as PI staining, which were based on nuclear changes. We treated approximately 8 × 105 U2OS and 6 × 105 HOS cells with experimental concentration range of HO-3867 for 24 h in one 6 cm plate. Following that, trypsinization was used to harvest viable cells along with floating nonviable cells. Following the protocol given by the manufacturer (BD Biosciences, San Jose, CA, USA), FITC Annexin V Apoptosis Detection Kit I was administered, followed by the analysis of the cell cycle through flow cytometry. Annexin V-FITC apoptosis staining was used in conjugation with PI staining to determine apoptosis from necrosis [29,30].

### 2.5. Human Apoptosis Array

To understand the effects of induced apoptosis, we followed the manufacturer’s protocols and used a Human Apoptosis Array Kit to define protein lysates from a vehicle-8 μM HO-3867-containing 2.4 × 106 U2OS cells that were treated for 24 h. In total, 35 proteins related to apoptosis were detected. The proteins were placed on a nitrocellulose membrane, detected with biotinylated detection antibodies, and then finally visualized through using chemiluminescent detection reagents.

### 2.6. Protein Extraction and Western Blot Analysis

We treated 8 × 105 U2OS and 6 × 105 HOS cells within a cell plate with experimental concentration range of HO-3867. The total cell lysates of U2OS and HOS cells were gathered and had their proteins extracted. We performed Western blot analysis using the primary antibodies against both uncleaved and cleaved forms of caspases 3, 8, and 9, as well as the antibodies for both unphosphorylated and phosphorylated forms of the MAPKs. For the antibody dilution, all antibodies were 1:1000 dilutions, except HO-1 antibody (1:5000 dilution). Horseradish peroxidase goat anti-rabbit and anti-mouse was then used for incubation before densitometry was used to measure the intensity [26,27]. After the intensity of each band was measured by densitometry, the relative intensities were calculated by normalizing to β-actin (1:1000 dilution; Santa Cruz Biotechnology, Inc., Dallas, TX, USA).

### 2.7. Statistical Analyses

The data from experiments went through statistical calculations performed by one-way analysis of variance (ANOVA) along with post hoc Tukey tests for more than two groups with equal sample sizes per group. Experiments were performed as independent and at least in triplicate experiments.

## 3. Results

### 3.1. HO-3867 Induces Cell Death in Human Osteosarcoma U2OS, HOS, and MG-63 Cells

**Figure 1:**
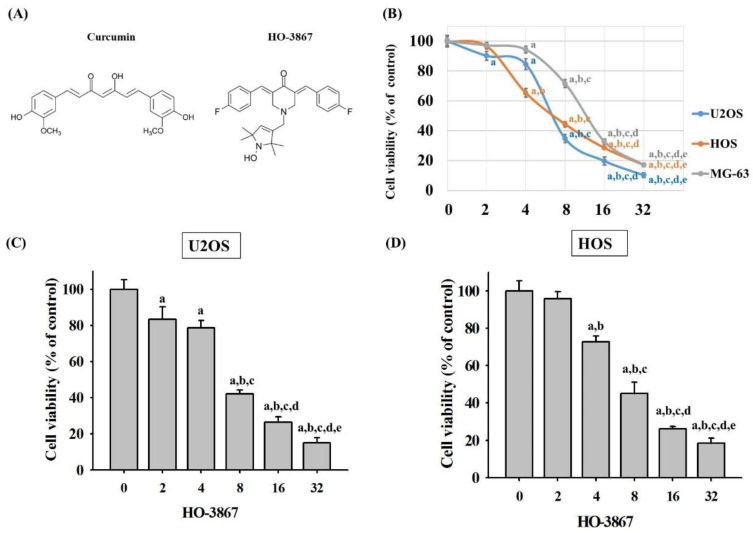
Analysis of cell viability in free and HO-3867-treated U2OS, HOS and MG-63 cells. (**A**) The chemical structures of curcumin and curcumin analog HO-3867 were drawn; (**B**) MTT assay was employed to detect the viability of U2OS, HOS, and MG-63 cells, which were treated with HO-3867 (2, 4, 8, 16, and 32 μM) for 24 h. After quantitative analysis, the effects are illustrated. *n* = 4. U2OS: *F* = 913.460, *p* < 0.001; HOS: *F* = 1110.912, *p* < 0.001; MG-63 (*n* ≥ 4): *F* = 880.549, *p* < 0.001; (**C**,**D**) CCk-8 assay was employed to detect the viability of U2OS and HOS cells, which were treated with HO-3867 (2, 4, 8, 16, and 32 μM) for 24 h. After quantitative analysis, the effects are illustrated. U2OS: *F* = 270.171, *p* < 0.001; HOS: *F* = 390.389, *p* < 0.001; ^a^ *p* < 0.05, when compared to 0 μM. ^b^ *p* < 0.05, when compared to 2 μM. ^c^ *p* < 0.05, when compared to 4 μM. ^d^ *p* < 0.05, when compared to 8 μM. ^e^ *p* < 0.05, when compared to 16 μM.

The chemical structures of curcumin and curcumin analog HO-3867 were drawn in Figure 1A. To define cytotoxicity of HO-3867 on osteosarcoma U2OS, HOS, and MG-63 cells, an MTT assay was performed. After treatment with HO-3867 for 24 h, U2OS, HOS, and MG-63 cells’ viability in concentrations of 2, 4, 8, 16, and 32 μM of HO-3867 was significantly unlike that of controls (0 μM) and showed dose-dependently (U2OS: *p* < 0.001; HOS: *p* < 0.001; MG-63: *p* < 0.001). (Figure 1B) After 24 h of HO-3867 (4, 8, and 16 μM) treatment, cytotoxicity in U2OS and HOS cells had dose-dependent increases, and their half maximal inhibitory concentrations (IC_50_) of HO-3867 were 6.91 μM in U2OS cells, 7.60 μM in HOS cells, and 12.24 μM in MG-63 cells. Moreover, cell proliferation was assessed by using the CCK-8 method in U2OS and HOS cells. As shown in Figure 1C,D, treatment of cells with HO-3867 for 24 h significantly decreased the proportion of viable cells in a concentration-dependent manner. Therefore, we picked the U2OS and HOS cell lines and used the experimental concentration range (0, 2, 4, 8, and 16 μM) for HO-3867 to explore its anticancer properties in the subsequent experiments.

### 3.2. HO-3867 Induces Cell Apoptosis and Arrest in the Sub-G1 Phase of U2OS and HOS Cells

**Figure 2:**
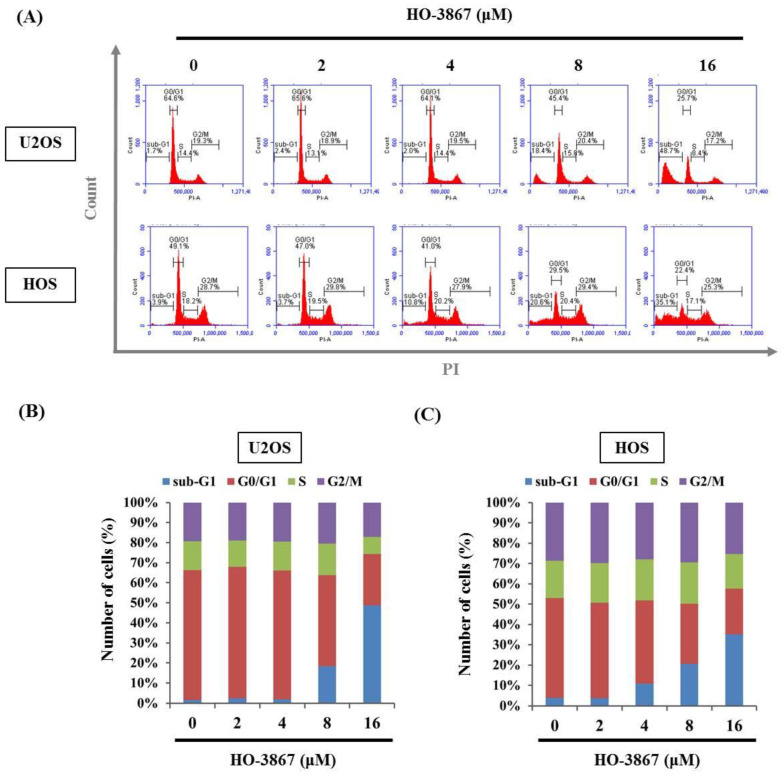
Analysis of cell cycle in HO-3867 treated U2OS and HOS cells. (**A**) After treatment with experimental concentration range of HO-3867 for 24 h, flow cytometry after PI was performed to determine DNA contents in U2OS and HOS cells; (**B**,**C**) The cell cycle profile of flow cytometry was subsequently quantified.

**Figure 3:**
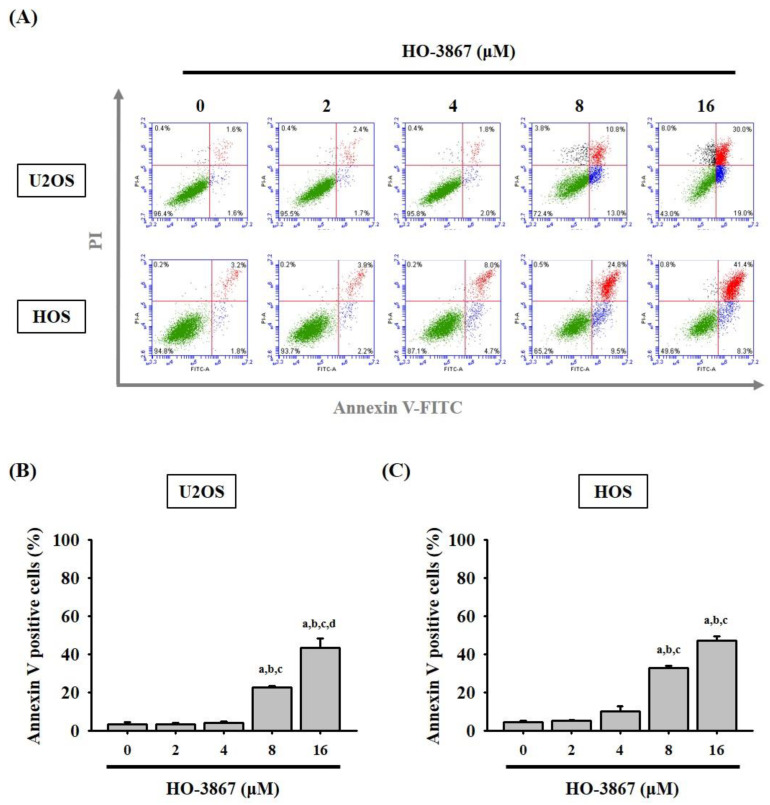
Analysis of cell apoptosis in HO-3867-treated U2OS and HOS cells. (**A**) After treatment with experimental concentration range of HO-3867 for 24 h, Annexin V-FITC/PI staining was performed to analyze DNA contents in U2OS and HOS cells. (**B**,**C**) The Annexin V-positive cells were subsequently quantified. U2OS: *n* = 6, *F* = 355.589, *p* < 0.001; HOS: *n* = 5, *F* = 18.887, *p* < 0.001. ^a^ Significantly different, *p* < 0.05, when compared to control. ^b^ Significantly different, *p* < 0.05, when compared to 2 μM. ^c^ Significantly different, *p* < 0.05, when compared to 4 μM. ^d^ Significantly different, *p* < 0.05, when compared to 8 μM.

To investigate the unknown mechanisms of HO-3867 inhibition of U2OS and HOS cell proliferation, flow cytometry was performed to examine the cell cycle. After being stained with PI, flow cytometry showed that 8 μM of HO-3867 drastically increased sub-G1 phase cell cycle accumulation from 1.7% to 48.7% in U2OS cells and 3.9% to 35.1% in HOS cells, suggesting that HO-3967 causes sub-G1 phase arrest in U2OS and HOS cells (Figure 2A–C). Additionally, detecting apoptosis at earlier stages, before gross morphological changes, is crucial for understanding the signaling pathways of programmed cell death. To verify whether or not the suppressive effects of HO-3867 on cell growth were caused by apoptosis and not others such as necrosis, Annexin V-FITC/PI apoptosis assay was used. Using both Annexin V-FITCH with PI staining, flow cytometry ensured that HO-3867 induced apoptosis of U2OS and HOS cells (Figure 3A,B). 

### 3.3. HO-3867 Increases the Cleaved Caspase 3 and Heme Oxygenase (HO)-1 Expression but Decreases XIAP and cIAP1 Expression in U2OS and HOS Cells

**Figure 4:**
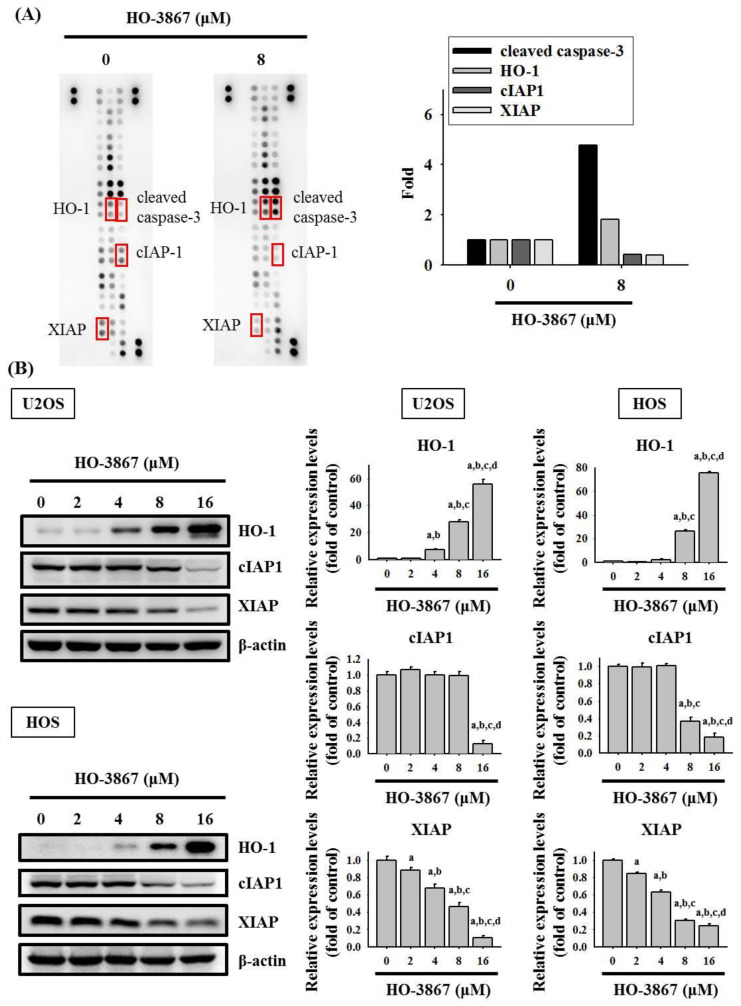
Analysis of the human apoptosis array and IAPs in HO-3867 treated U2OS cells. (**A**) After treatment with 8 μM of HO-3867 for 24 h in U2OS cells, the human apoptosis array was employed. The increased proteins cleaved caspase-3 and HO-1 as well as the decreased proteins cIAP-1 and XIAP were subjected to quantitative analysis; (**B**) After experimental concentration range of HO-3867 treatment for 24 h, Western blot analysis was performed to measure expressions of HO-1, cIAP-1, and XIAP in U2OS and HOS cells. Then, quantitative analysis was assessed. *n* = 3. HO-1: U2OS: *F* = 601.182, *p* < 0.001; HOS: *F* = 3705.167, *p* < 0.001; cIAP-1: U2OS: *F* = 227.563, *p* < 0.001; HOS: *F* = 864.074, *p* < 0.001; XIAP: *F* = 227.563, *p* < 0.001; HOS: *F* = 864.074, *p* < 0.001. ^a^ Significantly different, *p* < 0.05, when compared to control. ^b^ Significantly different, *p* < 0.05, when compared to 2 μM. ^c^ Significantly different, *p* < 0.05, when compared to 4 μM. ^d^ Significantly different, *p* < 0.05, when compared to 8 μM.

To demonstrate the mechanisms of apoptosis in U2OS cells caused by HO-3867, the human apoptosis array kit was used for determining apoptosis-related proteins. The human apoptosis array was performed on the U2OS cells that were treated with HO-3867 for 24 h, and the results showed increases in the cleaved caspase 3 and HO-1 proteins and decreases in cIAP1 and XIAP proteins (Figure 4A). To confirm the findings, Western blotting and quantitative analysis showed a significant increase in HO-1 (U2OS: *p* < 0.001; HOS: *p* < 0.001) but significant decreases in XIAP and cIAP1 in U2OS (XIAP: *p* < 0.001; cIAP1: *p* < 0.001) and HOS cells (XIAP: *p* < 0.001; cIAP1: *p* < 0.001) (Figure 4B).

**Figure 5:**
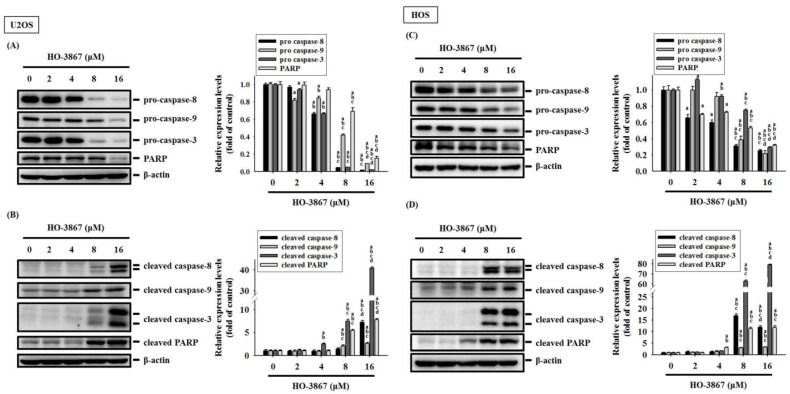
Analysis of activation of caspases 8, 9, and 3 in free and HO-3867-treated U2OS and HOS cells. After experimental concentration range of HO-3867 treatment for 24 h, Western blot analysis was performed to measure expressions of caspases 8, 9, and 3, and PARP as well as their active forms in (**A**,**B**) U2OS and (**C**,**D**) HOS cells. Then, quantitative analysis was assessed. *n* = 3. Caspase 8: U2OS: *F* = 4689.887, *p* < 0.001; HOS: *F* = 310.316, *p* < 0.001; caspase 9: U2OS: *F* = 1818.961, *p* < 0.001; HOS: *F* = 212.948, *p* < 0.001; caspase 3: U2OS: *F* = 7609.925, *p* < 0.001; HOS: *F* = 1718.653, *p* < 0.001; PARP: U2OS: *F* = 406.910, *p* < 0.001; HOS: *F* = 528.206, *p* < 0.001. Cleaved caspase 8: U2OS: *F* = 326.549, *p* = 0.01; HOS: *F* = 689.730, *p* < 0.001; cleaved caspase 9: U2OS: *F* = 36.155, *p* < 0.001; HOS: *F* = 96.503, *p* < 0.001; cleaved caspase 3: U2OS: *F* = 9138.156, *p* < 0.001; HOS: *F* = 10,315.365, *p* < 0.001; cleaved PARP: U2OS: *F* = 729.355, *p* < 0.001; HOS: *F* = 405.954, *p* < 0.001. ^a^ Significantly different, *p* < 0.05, when compared to control. ^b^ Significantly different, *p* < 0.05, when compared to 2 μM. ^c^ Significantly different, *p* < 0.05, when compared to 4 μM. ^d^ Significantly different, *p* < 0.05, when compared to 8 μM.

Within the tested proteins in the human apoptosis array, cleaved caspase 3 increased the most, meaning that the caspase 3 effector is the one responsible for dismantling U2OS and HOS cells. To identify the underlying effects of the caspase cascade caused by HO-3867, Western blotting was used to discover the effector caspase 3 as well as its upstream initiator caspases 8 and 9, and their cleaved forms. U2OS and HOS cells were then treated with experimental concentration range of HO-3867 for 24 h, the results dose-dependently showed fewer levels of pro-caspases 3, 8, and 9 dose-dependently (U2OS: *p* < 0.001; *p* < 0.001; *p* < 0.001; HOS: *p* < 0.001; *p* < 0.001; *p* < 0.001) and more expressions of cleaved caspases 3, 8, and 9 within higher concentrations (U2OS: *p* < 0.001; *p* < 0.001; *p* < 0.001; HOS: *p* < 0.001; *p* < 0.001; *p* < 0.001) (Figure 5A,B). We then discovered that HO-3867 activates extrinsic caspase 8 and intrinsic caspase 9 along with the downstream effector, caspase 3, to causes apoptosis in U2OS and HOS cells.

### 3.4. HO-3867 Activates Apoptotic Processes via the JNK-Signaling Pathway in U2OS and HOS Cells

**Figure 6:**
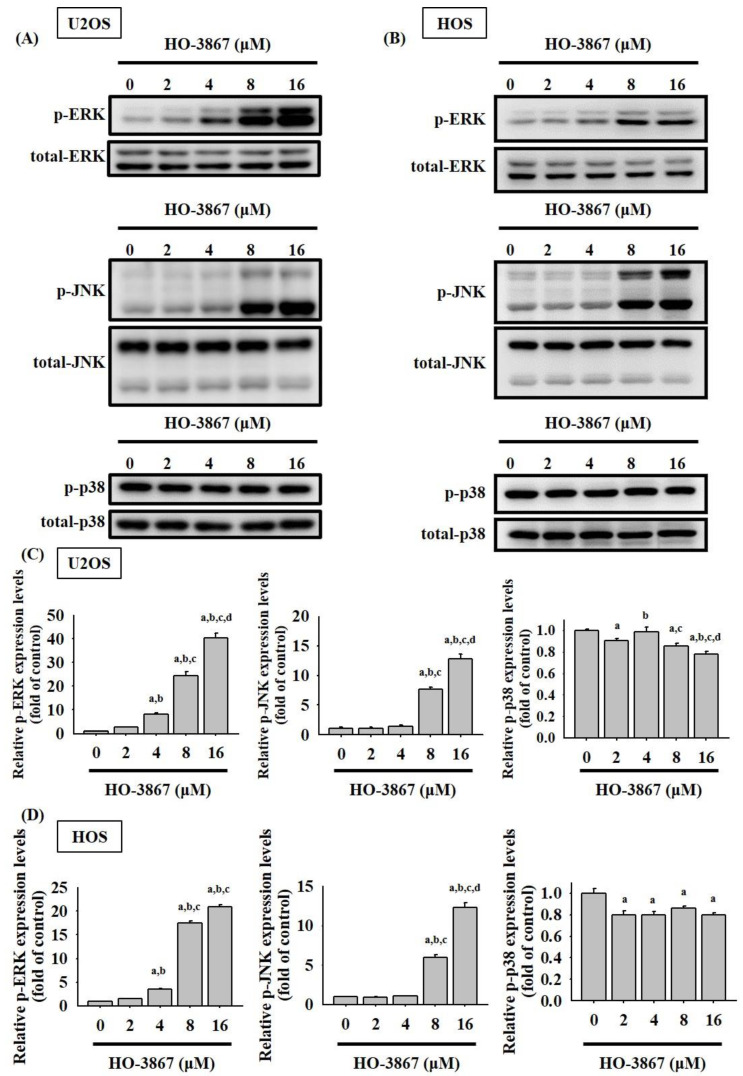
Analysis of phosphorylation of ERK1/2, JNK1/2, and p38 in HO-3867-treated cells. (**A**,**B**) After experimental concentration range of HO-3867 treatment for 24 h, Western blot analysis was performed to measure expressions of MAPKs, as well as their phosphorylation in (**A**) U2OS and (**B**) HOS cells. (**C**,**D**) Next, quantitative analysis was assessed. *n* = 3. *p*-ERK: U2OS: *F* = 661.501, *p* < 0.001; HOS: *F* = 4585.730, *p* < 0.001; *p*-JNK: U2OS: *F* = 494.446, *p* < 0.001; HOS: *F* = 855.033, *p* < 0.001; *p*-p38: U2OS: *F* = 33.591, *p* < 0.001; HOS: *F* = 23.845, *p* < 0.001. ^a^ Significantly different, *p* < 0.05, when compared to control. ^b^ Significantly different, *p* < 0.05, when compared to 2 μM. ^c^ Significantly different, *p* < 0.05, when compared to 4 μM. ^d^ Significantly different, *p* < 0.05, when compared to 8 μM.

MAPK pathways play an important role in regulating apoptosis by chemotherapeutic drugs as well as also being the upstream signaling of caspases 3, 8, and 9. To investigate further molecular mechanisms, Western blot analysis was then administered. As displayed in Figure 6A–D, HO-3867 was shown in increasing the phosphorylation of ERK 1/2 as well as JNK 1/2 dose-dependently within U2OS (*p* < 0.001; *p* < 0.001) and HOS cells (*p* < 0.001; *p* < 0.001), which indicates that HO-3867 activated the phosphorylation of the ERK 1/2 and JNK 1/2 pathways in the osteosarcoma cells. Yet, the phosphorylation of p38 showed inconsistent decreases after the application of HO-3867 in U2OS (*p* < 0.001) and HOS cells (*p* < 0.001).

**Figure 7:**
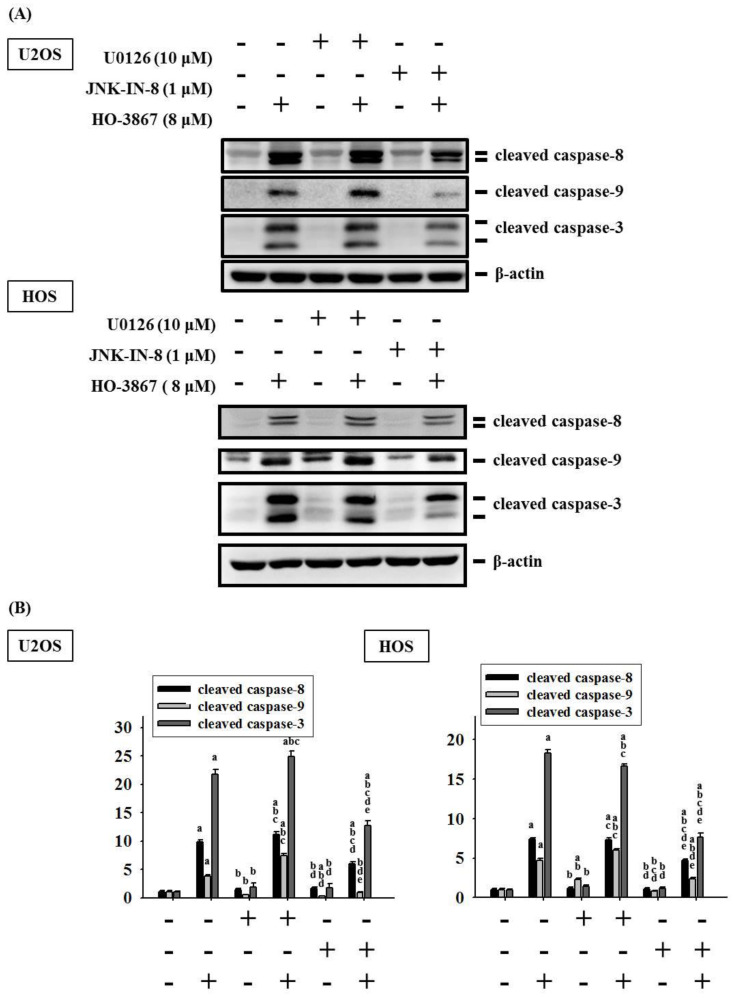
Analysis of HO-3867 on cleaved caspases 8, 9 and 3 expressions in HO-3867 with or without inhibitors of ERK1/2 (U0126)- and JNK1/2 (JNK-IN-8)-treated U2OS and HOS cells. (**A**) After pretreatment with or without 10 μM of U0126 and 1 μM of JNK-IN-8 for 1 h followed by 8 μM or no HO-3867 treatment for an additional 24 h, Western blot analysis was performed to measure expressions of cleaved caspases 3, 8, and 9 in U2OS and HOS cells; (**B**) Subsequently, quantitative analysis was assessed. *n* = 3. Cleaved caspase 8: U2OS: *F* = 536.028, *p* < 0.001; HOS: *F* = 719.244, *p* < 0.001; cleaved caspase 9: *F* = 550.879, *p* < 0.001; HOS: *F* = 436.343, *p* < 0.001; cleaved caspase 3: U2OS: *F* = 693.877, *p* < 0.001; HOS: *F* = 2338.671, *p* < 0.001. ^a^ Significantly different, *p* < 0.05, when compared to control. ^b^ Significantly different, *p* < 0.05, when compared to 8 μM HO-3867. ^c^ Significantly different, *p* < 0.05, when compared to U0126. ^d^ Significantly different, *p* < 0.05, when compared to U0126 and HO-3867 treatment. ^e^ Significantly different, *p* < 0.05, when compared to JNK-IN-8.

To identify whether the activated phosphorylation of ERK 1/2 and JNK 1/2 by HO-3867 would affect the intrinsic and extrinsic processes of U2OS and HOS cells through caspases 3, 8, and 9, a combination of the inhibitors of ERK 1/2 (U0126) and JNK 1/2 (JNK-IN-8) with or without treatment was used in Western blot analysis. Cleaved caspases 3, 8, and 9 were expectedly activated by 8 μM of HO-3867 (*p* < 0.001, *p* < 0.001, and *p* < 0.001) (Figure 7A,B). Additionally, the inhibitors of JNK1/2 significantly repressed the increase in cleaved caspases 3, 8, and 9 caused by the treatment of HO-3867 in U2OS and HOS cells. However, the inhibitor of ERK did not show suppressive effects on the increased levels of caspases 3, 8, and 9 caused by the treatment of HO-3867. These findings suggest that the JNK1/2 pathway is critical in the HO-3867-mediated apoptosis of extrinsic and intrinsic pathways as well as the downstream effector in U2OS and HOS cells.

## 4. Discussion

The high mortality and the main cause of most treatment failures rates of osteosarcoma is its highly metastatic potential [4,31] and failed chemotherapeutic treatments can result in the development of resistance within the tumor for future attempts of treatments [15,16,32]. Hence, to minimize the possibility of failed treatments, we conducted experiments and explored the underlying mechanisms of HO-3867, which is a synthesized curcumin analog for improving bioavailability compared to its original counterpart of curcumin, known for its apoptotic mechanisms at the molecular level [32]. Several studies have mentioned that curcumin is already considered to have a good anticancer effect [33,34]. In this study, we studied and demonstrated that HO-3867 possesses anticancer properties in human osteosarcoma.

HO-3867 has previously been known to cause apoptosis to cancer cells through the targeting of several key growth-regulatory proteins such as the Janus kinase (JAK) as well as STAT3 pathway to cause apoptosis among oral, ovarian, endometrial, and pancreatic cancers [35,36,37,38]. The compound has also been researched in terms of its ability to repress migration and invasion activity [39]. Moreover, HO-3867 has been shown to regulate the expression of FAS, FAK, and VEGF in order to suppress metastasis of ovarian carcinoma cells [40]. Overall, HO-3867 is versatile and has demonstrated an ability to initiate apoptosis or regulate metastasis in cancer cells by regulating various proteins in cancers.

Knowing from previous studies that HO-3867 is associated with apoptosis through various pathways in other cancer types, we focused on the effects of HO-3867 of various concentrations on various cell lines of human osteosarcoma. Results from flow cytometry showed that HO-3867 initiated apoptosis and decreased cell viability in human osteosarcoma U2OS, HOS, and MG-63 cells through the sub-G1 phase. As apoptosis can be initiated through various ways, we narrowed down the pathway after discovering an increase in HO-1 and a decrease in cIAP1 and XIAP. This is a crucial step in allowing us to identify that MAPK was utilized by HO-3867 for the results previously mentioned. Moreover, HO-3867 is widely considered as a selective STAT3 inhibitor [18,24]. Numerous studies have reported that STAT3 inhibitor exerts anticancer activity via different MAPK pathways [41,42,43]. However, the detailed mechanisms of how HO-3867 activates ERK 1/2 and diminishes *p*38 in U2OS and HOS cells still require further investigation.

Although *p53* is an important tumor suppressor gene, it is one of the most frequently mutated genes in cancer, implicated in more than half of all human cancers [44,45]. Mutant *p*53 (*p53^MT^*) loses the activity of wild-type *p*53 or expresses mutant proteins to inhibit the activity of the genome-guarding function through multiple mechanisms, which depend on different types of cancer and cell lines, even in the same cancer [44,45,46]. However, wild-type *p*53 (*p53^WT^*) is paradoxically retained in certain types or cell lines of cancers, such as *p53^WT^* U2OS and *p53^MT^* HOS cells [45,47]. HO-3867 covalently binds to mutant p53 to convert the mutant p53 protein to transcriptionally activate the wild-type *p*53 anticancer genetic response [44], whereas we interestingly found that HO-3867 suppressed p53 expression in both *p53^WT^* U2OS and *p53^MT^* HOS cells (Supplementary Figure S1). Whether p53 can regulate the antiapoptotic effect of HO-3867 by activating several targets, including *p*21, Slug, and Krüppel-like factor 4 (KLF4) in human osteosarcoma, should be extensively investigated.

To improve the bioavailability and potency of curcumin, the new synthetic curcuminoid HO-3867 was developed to target cytotoxicity toward cancer cells without influencing normal cells [18,25,26,36]. Using various experimental practices, we discovered that the addition of HO-3867 activates apoptotic processes via extrinsic and intrinsic pathways through an activation of the caspase cascade, IAPs, and the phosphorylation of MAPK pathways. Although the phosphorylation of ERK 1/2 and JNK 1/2 was observed in the study, we applied different combinations of inhibitors to confirm the exact pathway used by the analog to initiate apoptosis. Although the application of HO-3867 expectedly increased cleaved caspases 3, 8, 9, the additional application of the JNK inhibitor significantly repressed the increased values of the proteins while the co-treatment of the inhibitor of ERK did not show any suppressive effects. These findings suggest that the JNK-signaling pathway plays a critical role in the activation of apoptosis within the osteosarcoma U2OS and HOS cell lines through intrinsic and extrinsic processes, after treatment with HO-3867 but not through the ERK-signaling pathway. However, further studies are required to investigate whether the detailed results in vivo are similar to those in vitro and the positive efficacy of HO-3867 on human osteosarcoma could be obtained in clinical trials.

## 5. Conclusions

In conclusion, through our research and experiments, we investigated the anticancer efficacy of HO-3867 and investigated its mechanisms on human osteosarcoma cells. As shown by the results of the study, HO-3867 demonstrated its apoptotic mechanisms in human osteosarcoma; the combined results of HO-3867 on cancer from previous as well as the current study suggests the usefulness of HO-3867 for treating osteosarcoma.

## Data Availability

The data used to support the findings of the present study are available from the corresponding author upon request.

## Supplementary Materials

The following are available online at https://www.mdpi.com/article/10.3390/pharmaceutics14061257/s1, Figure S1: Analysis of p53 expression in HO-3867 treated U2OS and HOS cells.
