# Supplementary material for: HO-3867 Induces Apoptosis via the JNK Signaling Pathway in Human Osteosarcoma Cells
Source: Pharmaceutics. 2022 Jun 13;14(6):1257. doi: 10.3390/pharmaceutics14061257 (PMC9229449; doi:10.3390/pharmaceutics14061257)
Supplement: Supplementary file 1 [file pharmaceutics-14-01257-s001.zip › pharmaceutics-1739997-supplementary.pdf]

Supplementary Figure S1

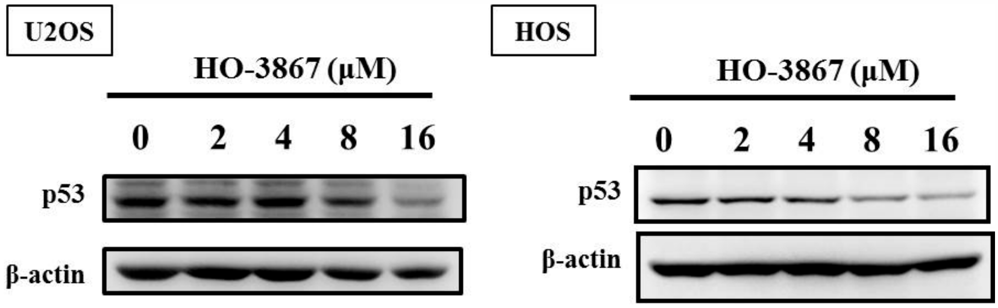

Supplementary Figure S1. Analysis of p53 expression in HO-3867 treated U2OS and HOS cells. After experimental concentration range of HO-3867 treatment for 24 h, Western blot analysis was performed to measure expressions of p53 in U2OS and HOS cells.
